# Heterogeneity of Developmental Dyscalculia: Cases with Different Deficit Profiles

**DOI:** 10.3389/fpsyg.2016.02000

**Published:** 2017-01-04

**Authors:** Ulf Träff, Linda Olsson, Rickard Östergren, Kenny Skagerlund

**Affiliations:** Behavioural Sciences and Learning, Linköping UniversityLinköping, Sweden

**Keywords:** developmental dyscalculia, symbolic number processing, non-symbolic number processing, time processing, spatial processing

## Abstract

Developmental Dyscalculia (DD) has long been thought to be a monolithic learning disorder that can be attributed to a specific neurocognitive dysfunction. However, recent research has increasingly recognized the heterogeneity of DD, where DD can be differentiated into subtypes in which the underlying cognitive deficits and neural dysfunctions may differ. The aim was to further understand the heterogeneity of developmental dyscalculia (DD) from a cognitive psychological perspective. Utilizing four children (8–9 year-old) we administered a comprehensive cognitive test battery that shed light on the cognitive-behavioral profile of each child. The children were compared against norm groups of aged-matched peers. Performance was then contrasted against predominant hypotheses of DD, which would also give insight into candidate neurocognitive correlates. Despite showing similar mathematical deficits, these children showed remarkable interindividual variability regarding cognitive profile and deficits. Two cases were consistent with the approximate number system deficit account and also the general magnitude-processing deficit account. These cases showed indications of having domain-general deficits as well. One case had an access deficit in combination with a general cognitive deficit. One case suffered from general cognitive deficits only. The results showed that DD cannot be attributed to a single explanatory factor. These findings support a multiple deficits account of DD and suggest that some cases have multiple deficits, whereas other cases have a single deficit. We discuss a previously proposed distinction between primary DD and secondary DD, and suggest hypotheses of dysfunctional neurocognitive correlates responsible for the displayed deficits.

## Introduction

Increasingly more attention is being directed toward identifying the neurocognitive profile and origins of developmental dyscalculia (DD), a specific learning disorder characterized by severe impairments in acquiring mathematical competency (American Psychiatric Association, 1994). Important strides have been made and different single core deficit hypotheses have been proposed. Each hypothesis has received some empirical support from both behavioral and neuroimaging studies.

It has been known for quite some time that children show different kinds of mathematical difficulties (Geary, 1993). Thus, it is increasingly recognized that DD is heterogeneous and the observed phenotype(s) might be caused by a multitude of underlying neurocognitive causal factors (Rubinsten and Henik, 2009; Kaufmann et al., 2013; Skagerlund and Träff, 2016). Determining these specific causal factors is further complicated by frequent comorbidities, such as ADHD or dyslexia (Von Aster and Shalev, 2007). Consequently, some researchers propose that the scientific community should differentiate between primary and secondary DD (Kaufmann et al., 2013; Price and Ansari, 2013). Primary DD is characterized by a severe deficit in numerical or arithmetic functioning, caused by different underlying biological factors. Secondary DD denotes individuals whose impaired numerical capacity can be explained entirely by non-numerical impairments, such as attention or working-memory processes (Kaufmann et al., 2013). Thus, even primary DD may be subject to further subtyping, dependent on different underlying factors.

In order to implement appropriate educational interventions, it becomes crucial to identify the subtypes and their underlying causes. Therefore, not only behavioral measures such as test scores, error rates and response times should be used to describe DD on a group level. By using a case study methodology, we can go beyond summary statistics and instead investigate individual idiosyncrasies on a cognitive level. To identify the neural microstructures and genetic dispositions causing the observed phenotypes, several levels of analyses should be investigated, such as the behavioral, cognitive, and neural level. In this way we can create a multilevel taxonomy of DD, which can also be used to guide further neuroimaging studies.

In the current study, four children with homogenous mathematical-behavioral profiles fitting the characteristics of DD were selected to provide a clear demonstration of the heterogeneity of DD. The purpose was to investigate the cognitive and number processing skills of the four children and relate their behavioral profile to predominate hypotheses. To this end, three levels of description were used: (1) At the behavioral level, four children were identified suffering from DD (2) at the cognitive level, a comprehensive test battery was administered to evaluate the cognitive profile and performance, (3) at the neurocognitive level, the results of the above mentioned levels were used to hypothesize about the underlying neurocognitive correlates with the purpose of guiding future neuroimaging studies.

## Core hypotheses regarding DD

A predominant hypothesis is that DD (see Table 1 for an overview of different hypotheses of DD) originates from a core deficit in the innate *Approximate Number System* (ANS), which enables humans to represent quantities in an approximate manner. It has been suggested that this system constitutes the foundation onto which the symbolic number system is mapped (Gelman and Butterworth, 2005; Piazza, 2010; Dehaene, 2011). Individual ANS acuity can be measured by having participants determine which of two simultaneously—and briefly—presented sets of objects is more numerous. Using psychophysical modeling, given the assumption that the ANS operates according to Weber's law, individual Weber fractions (*w*) can be used as an index of individual ANS acuity (Mazzocco et al., 2011a). ANS acuity has been found to be related to mathematical proficiency (Gilmore et al., 2010; Mazzocco et al., 2011b) and studies have found that children with DD have poorer ANS acuity than their typically achieving peers (Landerl et al., 2009; Piazza et al., 2010; Mazzocco et al., 2011a; Mejias et al., 2012; Skagerlund and Träff, 2016).

**Table 1:** **An overview of the different hypotheses of developmental dyscalculia**.

**Hypothesis**	**Cognitive signature**	**Site of neurocognitive dysfunction**	**Example of research**
ANS deficit	Deficit in approximating and representing quantities	IPS morphology and hypoactivation	Piazza et al., 2010; Mazzocco et al., 2011a
Numerosity coding deficit	Impaired exact counting of quantities	IPS morphology and hypoactivation	Zorzi et al., 2005; Butterworth, 2010
Magnitude processing deficit	Deficit in processing analog magnitudes	IPS morphology and hypoactivation	Vicario et al., 2012; Skagerlund and Träff, 2014
Access deficit	A disconnect between the symbols and quantity representations	IPS and AG/IFG disconnection	Rousselle and Noël, 2007; De Smedt and Gilmore, 2011
Working-memory deficit	Impaired working-memory capacity	Prefrontal cortex	Geary, 1993; Andersson and Lyxell, 2007
Executive functioning deficit	Impaired shifting ability	Prefrontal cortex	van der Sluis et al., 2004; Szucs et al., 2013

Butterworth (2010; Zorzi et al., 2005) present a second core deficit account of DD, the numerosity-coding hypothesis. It posits that DD is due to a deficit in the internal number code that represents each quantity exactly as a set of discrete elements within and above the small number range.

Neuroimaging studies have begun to map the neurocognitive correlates of number processing and arithmetic. Research indicates involvement of the intraparietal sulcus (IPS) during non-symbolic number processing (e.g., during ANS tasks) and arithmetic calculations (Gruber et al., 2001). Furthermore, children with DD have both functional and structural abnormalities in this region in terms of gray matter volume and activation patterns (Price et al., 2007; Kaufmann et al., 2011; Ashkenazi et al., 2012). Further support for the involvement and importance of the IPS comes from a recent study by Iuculano and Cohen Kadosh (2014). By using transcranial stimulation of the posterior parietal cortex of an individual diagnosed with DD, they demonstrated that this individual showed improved numerical proficiency. This highlights the role of the parietal cortex in number processing (Iuculano and Cohen Kadosh, 2014).

Recent studies indicate that children with DD or mathematical difficulties not only have number processing deficits but also problems in processing other magnitudes such as time and space (Ashkenazi and Henik, 2010; Vicario et al., 2012; Moll et al., 2014; Skagerlund and Träff, 2014). For example, Skagerlund and Träff (2014) found that 10-years olds with DD showed impaired ANS acuity (i.e., non-symbolic number discrimination), but also problems with time discrimination and difficulties with two spatial skills; spatial visualization (paper-folding) and mental rotation. The results support the “*A Theory Of Magnitude”* (ATOM) *model* (Walsh, 2003; Bueti and Walsh, 2009), which states that time, space and number are processed by a partly shared general magnitude system. However, each dimension is also supported by dimension-specific processes (Walsh, 2003; Cantlon et al., 2009; Cappelletti et al., 2009). Strong evidence for a shared magnitude system is that time and space display the typical effects of distance, size and *SNARC (Spatial Numerical Association of Response Codes*; Dehaene et al., 1993) effect consistently found for numbers. The distance effect refers to the fact that the selection of the larger of two numerals is performed faster when the numerical distance is large (1 vs. 9) compared to when it is small (1 vs. 2; Moyer and Landauer, 1967). The fact that comparing numbers becomes increasingly difficult the larger they are, even when the distance between them is kept constant (e.g., comparing 8 and 9 is more difficult than comparing 2 and 3) constitutes the classical problem-size effect. The SNARC effect entails faster left-sided responses to smaller numbers and faster right sided responses to larger numbers. These three effects are considered to demonstrate that numbers are spatially represented as approximate analog magnitudes (i.e., mental number line) in an ascending left to right order, that are automatically accessed when numerical information is processed. The distance effect has been shown for many different non-numerical magnitude stimuli such as area (e.g., Fulbright et al., 2003), length, time (Dormal et al., 2006), and pitch (Rusconi et al., 2006). The size effect has also been observed with non-numerical magnitude stimuli, for example, Fias et al. (2003) obtained a size effect when subjects compared pairs of angles or pairs of lines. The same is true for the SNARC effect, for example, (Ishihara et al. (2008); see also Vicario et al., 2008) demonstrated that time is also spatially represented, resulting in the so-called STEARC effect (Spatial-Temporal Association of Response Codes). Another line of evidence for a shared magnitude system has been provided by experimental studies examining the interaction between magnitudes. A number of studies demonstrate bidirectional influence between space and number, (see Bueti and Walsh, 2009; see also Chang et al., 2011). Similar influence has been found between number and time, but with time processing more often affected by number processing than vice versa (e.g., Cappelletti et al., 2011). Also space and time have demonstrated to exert reciprocal influence on each other (Ishihara et al., 2008; Fabbri et al., 2012).

(Skagerlund and Träff, 2014) results were also in accordance with Feigenson (2007), who suggested that if the diverse magnitude representations share a common mechanism, deficits in one dimension should be paralleled by deficits in other magnitude processing abilities. This led Skagerlund and Träff (2014) to suggest that children with DD, whose primary deficit was thought to be circumscribed to the ANS prior to the study, may in fact suffer from a more comprehensive magnitude processing deficit that extends from quantity processing to also include processing of time and space.

Humans are also believed to be equipped with a second system involved in number processing, called the *object-tracking system* (OTS) (Wilson and Dehaene, 2007; Piazza, 2010; Piazza et al., 2010; Dehaene, 2011), a visuospatial object-based attention system for keeping track of 3–4 objects exactly. One characteristic of the OTS is that it allows effortless and quick apprehension of 1–4 objects, called subitzing. Earlier research indicates that children with DD have a restricted subitizing range of a maximum of 3 objects (van der Sluis et al., 2004; Desoete and Grégoire, 2006; Moeller et al., 2009; Andersson and Östergren, 2012), whereas typically developing children can quickly apprehend up to 4 objects (Ashkenazi et al., 2013).

*The access deficit hypothesis* (Rousselle and Noël, 2007) states that DD is caused by a defective connection between the symbols (e.g., digits) and the underlying magnitude representations. Thus, DD is not due to a deficit in the innate ANS *per se* (Rousselle and Noël, 2007; see also Wilson and Dehaene, 2007). Evidence has been reported by De Smedt and Gilmore (2011), Landerl and Kölle (2009), and Rousselle and Noël (2007) as children with DD displayed problems with symbolic number comparison, but normal performance on non-symbolic number comparison. Although Skagerlund and Träff (2016) found a subgroup of children with DD showed poor ANS acuity, another subgroup was also identified. They showed intact ANS', but had difficulties in accessing the underlying semantic representation from symbols. This led the authors to suggest that DD is heterogeneous disability with different subtypes, including one characterized by an access deficit (Skagerlund and Träff, 2016).

Recent neuroimaging studies have concluded that DD can best be described as a disconnection syndrome (Rykhlevskaia et al., 2009; Ranpura et al., 2013) in which the primary neurocognitive biomarker can likely be traced to an aberrant connectivity pattern between brain regions. These neuroimaging studies have used diffusion tensor imaging (DTI) to explore white matter integrity in the brain in individuals with and without DD. White matter development is an important aspect of brain maturation, reflecting connectivity between brain areas, and it is associated with learning (Ranpura et al., 2013). For example, Rykhlevskaia et al. (2009) found that their participants exhibited abnormal temporal-parietal white matter. A network analysis led the researchers to suggest that DD is a disconnection syndrome (see also Kucian et al., 2014). Another study focused on the developmental trajectory of gray and white matter, where DD children did not show increases in white matter in the frontal lobes nor in the parietal lobes as a function of age, which could be observed in controls (Ranpura et al., 2013). This may suggest that the frontal lobes do not connect adequately to the parietal lobes during ontogenetic development. The parietal lobes consist of key structures, such as the IPS and the angular gyrus (AG). The AG is believed to be involved during retrieval of arithmetical facts and semantic processing of numerical symbols (Ansari, 2008; Ranpura et al., 2013). These neuroimaging studies provide support for the notion that DD is, at least partly, a disconnection syndrome. These individuals with DD may struggle with the retrieval of arithmetical facts and accessing the non-symbolic magnitude representations even if the representations themselves, and the cortical loci subserving them, are intact. Thus, these findings may be compatible with the access deficit hypothesis, although this needs to be empirically verified.

The domain-general cognitive deficit hypothesis is a fundamentally different account of DD, postulating that deficits in the underlying domain-general cognitive system impede the development of age-adequate mathematical skills (e.g., Geary, 1993; Geary and Hoard, 2005). Numerous studies have found that children with DD display domain-general cognitive deficits (e.g., working memory, executive functions, processing speed; Bull et al., 1999; Swanson and Beebe-Frankenberger, 2004; van der Sluis et al., 2004; Andersson and Lyxell, 2007; Passolunghi and Cornoldi, 2008; D'Amico and Passolunghi, 2009; Andersson, 2010; Raghubar et al., 2010). These findings in the view of primary and secondary DD, suggest that these children have secondary DD (Kaufmann et al., 2013; Price and Ansari, 2013).

To summarize, it is clear from previous studies that have used group comparisons that the origin of DD is multifactorial and not solely caused by a core deficit (Kosc, 1974; Jordan et al., 2002; Mazzocco and Myers, 2003; Dowker, 2005; Wilson and Dehaene, 2007; Rubinsten and Henik, 2009). In fact, Andersson and Östergren (2012) obtained data consistent with three core deficit accounts (defective ANS; ANS and OTS; domain-general cognitive deficits) in one sample of children. The results are in favor of the multiple deficits account rather than a core deficit account.

## Developmental dyscalculia as a heterogeneous neurocognitive disorder

The multiple deficits account is reasonable considering that prior studies have all used group comparisons. A flaw of this research method is the lack of sensitivity to the variability among individuals with DD; although no significant group differences may emerge on a particular task, some DD participants still display severe difficulties. Conversely, some DD participants may display adequate performance on tasks that show significant group differences. Thus, only relying on traditional group comparisons might not be the optimal design for exploring the heterogeneity of a phenomenon such as DD. Collapsing all individuals into a single, supposedly, homogeneous group may obfuscate important individual variability. In this paper, four cases of DD are analyzed to further understand the heterogeneity regarding the origin(s) of DD.

Although the bulk of research on DD has ignored its heterogeneity, there are some recent exceptions. Bartelet et al. (2014) examined a sample of 226 children (grade 3–6) with DD on a comprehensive test battery of number processing and domain-general cognitive tasks and used cluster analysis to identify subgroups of DD with different cognitive profiles. The cluster analysis generated six clusters; Cluster 1 was characterized by problems with the number line estimation task but no other problems. Cluster 2 was characterized by problems with the approximate numerical knowledge and number line estimation tasks. Cluster 3 displayed the same problems as cluster 2 but also weak spatial short-term working memory. Cluster 4 was distinguished by weak symbolic number processing. Cluster 5 did not display any number-specific cognitive processing weaknesses, additionally having a strong verbal short-term working memory. Cluster 6 was characterized by low non-verbal IQ, but no other problems. The clusters support the notion that DD is a heterogeneous disorder with a multifactorial origin.

In view of the core deficit hypotheses, cluster 2 and 3 correspond to the defective ANS hypothesis, whereas cluster 4 is consistent with the access deficit hypothesis. Cluster 1 demonstrates that some children with DD have specific problems in developing an accurate symbolic number line, which can be considered a higher level of number processing (cf. Von Aster and Shalev, 2007). In contrast, none of the clusters were in line with the hypothesis of domain-general cognitive deficits only. Furthermore, cluster 5 suggests (still) unexplored origins of DD. A tentative interpretation is that the arithmetic impairment in this subgroup can be attributed to exogenous factors, such as poor motivation or education (Bartelet et al., 2014).

Further support for the multiple cognitive deficits hypothesis is provided by Skagerlund and Träff (2016), as they examined children with different profiles of mathematical deficits. They found that children with calculation and arithmetic fact retrieval problems suffered from an impairment of the ANS, whereas children with only arithmetic fact retrieval problems suffered from an access deficit.

A few researchers have used case studies to examine the heterogeneity of DD. Iuculano et al. (2008) studied two boys with DD. Neither case demonstrated problems with non-symbolic approximate number processing, excluding the possibility of a defective ANS. The first case's results were consistent with the access deficit hypothesis, as performance was poor on the symbolic number comparison task. The second case displayed weak dot enumeration performance, but performed at normal levels on all other number tasks. This performance pattern suggested a deficit with processing exact numerosities, in accordance with the defective numerosity-coding hypothesis (Zorzi et al., 2005; Butterworth, 2010).

Another case study was DB, a 42 year old woman with good overall cognitive capacities but who suffered from a severe deficit in arithmetic fact retrieval (De Visscher and Noël, 2013). DB's results suggested an intact ANS as well as intact access to it from symbols. However, extensive testing of her long-term memory indicated a hypersensitivity-to-interference, preventing DB from establishing an arithmetic facts network.

In sum, prior studies reveals that three different lines of research provide convergent evidence that DD originates from multiple deficits: Traditional studies making group comparisons by testing and contrasting different core deficit accounts, studies aiming at identifying subgroups with different deficit profiles by making group comparisons, and case studies aiming at identifying the full spectrum of heterogeneity regarding the origin(s) of DD.

## The present study

The aim of the present paper was to expand our knowledge regarding the origin(s) of DD, by testing the main core deficit accounts previously described in relation to four cases of DD with similar profiles of mathematical deficits. The ATOM hypothesis, that a general magnitude-processing deficit underlies DD, was also tested (cf. Skagerlund and Träff, 2014).

The main research question was whether all four cases displayed similar cognitive deficit profiles consistent with the same or different core deficit accounts. The latter outcome would suggest that DD originates from multiple deficits.

## Methods

### Case descriptions

The four cases consist of three second-graders and one third-grader. The four cases are termed C1 (boy, 8 years, 4 months), C2 (girl, 9 years, 5 months), C3 (girl, 8 years, 7 months), and C4 (boy, 8 years, 2 months). Swedish was their native language, and they had normal or corrected-to-normal visual acuity and no hearing loss. The four cases were selected based on four criteria: (1) The child should have received special education in mathematics at the time of and at least one semester prior to the study. (2) The child's score on three of the five arithmetic measures (see below) had to be at or below the 10th percentile (i.e., at or above *z*-score of −1.29) of the norms. (3) The child should not have any history of neurologically based impairments, such as ADHD or other known learning disabilities (e.g., dyslexia), neither were they subject to assessment of ADHD or other learning disabilities. (4) To exclude poor schooling and general intellectual impairments as underlying causes of low mathematical performance, the child's score on standardized reading tests and Raven's Standard Progressive Matrices (see below) had to be above the 15th percentile (i.e., above *z*-score of −1.00) of the norms. The four cases' raw scores (*z*-scores) on Raven's, reading, and arithmetic tasks, along with mean performance (SD) for the norm groups are presented in Table 2.

**Table 2:** **Performance (*z*-scores) of the four cases of DD and mean performance (SD) of the norm groups on Raven's, reading and arithmetic**.

**Task**	**C1 Boy grade 2**	**C2 Girl grade 3**	**C3 Girl grade 2**	**C4 Boy grade 2**	**Norms grade 3**	**Norms grade 2**
Ravens	17 (–0.38)	18 (–0.98)	18 (–0.26)	16 (–0.51)	24.06 (6.11)	20.17 (8.24)
Reading comprehension	6 (–0.31)	8 (–0.48)	7 (–0.06)	5 (–0.56)	10.22 (4.26)	7.24 (3.98)
Word-decoding	106 (0.77)	88 (–0.60)	113 (0.98)	110 (0.89)	105 (28.50)	80.50 (33.00)
Calculation	0 (–1.29)	4 (–0.55)	0 (–1.29)	0 (–1.29)	5.36 (2.49)	2.96 (2.29)
Equation	1 (–1.12)	0 (–3.03)	1 (–1.12)	0 (–1.51)	7.36 (2.43)	3.86 (2.55)
Arithmetic fluency	3 (–2.10)	6 (–1.94)	11 (–1.34)	9 (–1.53)	32.18 (13.48)	25.18 (10.58)
Number facts (3 s)	0 (–0.94)	0 (–1.46)	0 (–0.94)	0 (–0.94)	10.09 (6.93)	5.62 (5.99)
Number facts (> 3 s)	2 (–11.48)	17^*^ (–3.17)	2 (–11.48)	20^*^ (–0.89)	22.71 (1.80)	21.51 (1.70)

* *Finger counting*.

All cases performed within the normal range on Raven's, the two reading tasks, as their *z*-scores were above –1.00. None of them had circumscribed problems with a specific mathematical skill, such as arithmetical fact retrieval, a proposed subtype of DD (De Visscher and Noël, 2013; Skagerlund and Träff, 2016), but rather show pervasive mathematical difficulties across several aspects of mathematics.

The C1 case performed at/below the cut-off criterion of *z* = −1.29 on three (calculation; arithmetic fluency; number facts > 3 s) of the five arithmetic measures. The C2 case performed below the cut-off criterion on all measures except for the calculation task. A distinctive feature of her skills is that she used a finger counting when solving simple single-digit arithmetic problems (number facts > 3 s). Despite this strategy, she scored considerably below the norm group mean on the number facts measure (> 3 s). The C3 case displayed severe problems with the calculation task, the arithmetic fluency and the number facts (> 3 s) measure. Her performance was also weak on the equation, and the number facts (< 3 s) measures. The C4 case showed impaired performance on the calculation, the equation, and the arithmetic fluency tasks, whereas his performance on the number facts (> 3 s) measure was normal. This distinction in performance is probably due to C4's use of finger counting when solving single-digit problems.

### Norm groups

Four independent and unselected groups were used as norms for the case in third grade. The sample sizes of the four groups were 53 (equation and shifting task), 145 (word-decoding), 115 (visual working memory, time discrimination; number naming), and 274–292 for all other tasks. Three independent and unselected groups were used as norms for the three cases in second grade. The sample sizes of the three groups were 164 (word-decoding), 292–303 (reading comprehension, calculation, addition fluency, verbal working memory, color naming; mental rotation) and 66 for all other tasks. All children in the norm groups reported having Swedish as their native language, normal or corrected-to-normal visual acuity, and no hearing loss.

### Measures

A comprehensive test battery was administered, tapping basic mathematical and reading skills, fluid intelligence (Raven's Standard Progressive Matrices, Raven, 1976), and domain-general cognitive abilities (e.g., working memory). Tasks tapping magnitude processing (number, temporal, spatial) were also included. The selection of tasks was based on current hypotheses regarding the origin of DD.

#### Raven's standard progressive matrices

This test of fluid intelligence is well-known and frequently used. It consists of a series of visual pattern designs with a piece missing, where the child selects which of six-eight options displayed beneath the design is the missing piece. The test includes five sets of designs (A, B, C, D, E), with 12 items per set. Only sets B, C, and D were used in this study. The child received a test booklet, and after two demonstration/practice items had been performed with the experimenter, the child individually completed the 36 items at her/his own pace. The number of correctly answered problems was used as the dependent measure of fluid intelligence.

#### Reading comprehension

This test consisted of a short story read by the child (Malmquist, 1977). The narrative took the form of a fairy-tale, and scattered throughout the text were single missing words replaced by a blank space and a bracket containing four words. The child then had to select which of the words made the most sense in terms of the sentence and the story, and underlined their answer. This reading test contained 20 items (i.e., missing words) scattered evenly throughout the story. The number of correct items selected within 4 min was the dependent measure.

#### Reading (word-decoding)

In this task (Elwér et al., 2009), the child read as many words as possible from a list of 100 words, presented in four columns with 25 words in each column, during 45 s. The child was instructed to read as quickly as possible without making any errors. The experimenter used a stopwatch to keep track of time, continually checked the child's answers and registered each error. The task consisted of two sheets of paper, A- and B-version, and the child performed both versions beginning with the A-version. The combined number of correctly read words from the two versions was used as the dependent measure.

#### Arithmetic calculation

Using three pen-and-paper tasks, calculation ability was tapped. The items were designed to become increasingly difficult. The same procedure (i.e., instructions, paper and pencil, scoring procedure) was used on all three subtasks. In the first calculation subtask, the child was asked to solve six addition and six subtraction problems (e.g., 57+42; 78–43; 568+421; 658–437) in 8 min. The problems were presented horizontally, and the child responded in writing. All problems, except two, involved regrouping (i.e., carrying or borrowing). The child could solve the problems in any way according to their own preference. However, only paper and pencil was at their disposal. The total number of solved problems was used as the dependent measure of calculation ability.

#### Arithmetic equations

The tasks consisted of 12 equations presented horizontally (e.g., 61 + ___ = 73; ___ + 25 = 500), where the child had to enter the correct digit so the equation was correct. The child was allowed 7 min to perform the task. The number of correctly solved equations, out of 12, was the dependent variable.

#### Arithmetic fluency

The task was to solve as many single-digit addition (e.g., 2 + 5) and subtraction (e.g. 6–2) problems as possible during two separate 60 s trials. The task consisted of two sheets of paper, an addition and a subtraction version, containing 81 problems presented in three columns. The experimenter used a stopwatch to keep track of time. The number of correctly solved problems was used as the dependent variable.

#### Arithmetic fact retrieval

This task was computer administrated and consisted of 12 addition (e.g., 9 + 5; 4 + 6) and 12 subtraction problems (8–4; 6–2) administered in two separate blocks. One problem at a time was presented horizontally on the computer screen. When the child was ready, the experimenter pressed the mouse button, and a problem was displayed on the screen until the child had responded. A timer started at the onset of the problem and was stopped when the experimenter pressed the mouse button after the child had given an oral response to the problem. The child was instructed to provide an answer immediately by remembering what the answer is and was encouraged to guess if he/she failed to do so. Two different measures were used: (1) number of correctly solved problems with response times within 3 s, (2) number of correctly solved problems including response times longer than 3 s (cf. Russell and Ginsburg, 1984). Use of finger counting was also registered.

#### Complex word repetition

In this verbal working memory task, the child was presented with word sequences. The task was to decide whether each presented word was an animal or not by answering “yes” or “no” (no animal, e.g., car), before the next word was presented. At the end of the sequence, the child had to recall the words in correct (serial) order. The first span size employed was two words, the next was three, and so forth. Two trials were presented for each span size. All children were asked to complete up to span size four, regardless of whether the correct order was recalled. However, if the child managed to recall the correct serial order beyond span size four testing continued until the child failed both trials of the same span length. Half of the words in the sequences were animals. Verbal working memory span was measured as the longest sequence remembered correctly (in serial order), plus 0.5 points if the child managed to recall all trials correctly on the same span size.

#### Visual matrix task

The participant sat in front of a computer screen and was initially presented with a 3x3 matrix of empty squares. After 1000 ms two black dots appeared in one of the squares. The first task was to decide whether these two dots were of equal size, and press the “*” key if they were equal or the “A” key if they were not. The child had 3 s to respond, after which two additional dots appeared in another square while the former two dots were still visible. The second, and main, task was to remember the location of the dots in the matrix. When a predetermined number of dots had appeared, which depended on the current difficulty level, the matrix and the dots disappeared from the screen. The child was then given a sheet of paper with an empty matrix depicted on it. The child was then asked to mark the squares in which the dots had been presented. The first matrix had 3 × 3 squares in which two squares contained black dots (i.e., span size two). The next matrix had 3 × 4 squares in which three squares ultimately were filled with dots (i.e., span size three). In this way, the complexity of the matrices increased for each new span size. The child was given two trials for each span size. Testing stopped when the child failed both trials within a given span size, otherwise the testing continued. Visuospatial working memory span was measured as the longest sequence of dots remembered correctly (in serial order), plus 0.5 points if the child managed to recall all trials correctly on the same span size.

#### Trail-making

Cognitive shifting was assessed using a paper-and-pencil version of the Trail Making Test (McLean and Hitch, 1999; van der Sluis et al., 2004), composed of two conditions. The first condition (A) consisted of 22 circles, each containing a digit, whereas the second condition's (B) 22 circles contained either a digit or a letter. In condition A, the task was to draw a line between the circles in ascending order as quickly as possible. In condition B, the children were again told to draw the line and connect the circles in ascending order as fast as possible, but now in alternating order (1-A-2-B-3-C etc.). Seconds needed to complete each condition was used as the dependent measure. Shifting ability was assessed by subtracting the completion time of condition A from B.

#### Color naming

This task was administered on two sheets of paper, where 30 “XXX”s (Arial, 22-point font) were printed in different colors (red, green, blue, black, and yellow), in two columns. The child was instructed to name the printed color of the XXX's as fast as possible, without making any errors. A stopwatch was used to measure the total response time used as the performance measure. The combined response times for the two sheets of paper were used as a measure of speed of access to semantic information in long-term memory.

#### Number naming

The task was administered on two sheets of paper. The single-digit condition consisted of seven rows of the digits 1–9 printed in black ink. Each digit appeared once per row, resulting in 63. The double-digit condition consisted of six rows and 27 digits, each appearing twice. The participant was told to name each digit as fast as possible, without making any errors. A stopwatch was used to measure the total time needed to name all digits. All children began with the single-digit condition. The combined response time for the single- and double-digit conditions was used as the dependent measure.

#### Non-symbolic number comparison

The task was administrated via the Panamath software program (v. 1.21), developed by Halberda and Feigenson (2008). Two arrays were presented, containing between 5–21 blue and yellow dots for 1506 ms. The child had to decide which array was more numerous, and then press the key corresponding to the appropriate side of the screen (F- or L-key). The child had an unlimited amount of time to indicate their response and pressed the space bar to enable the next trial. Prior to each trial, a fixation cross was displayed on the center of the screen. Four ratios (1.24; 1.37; 1.60; 2.60) were presented 26 times each, yielding a total of 104 trials. Two practice trials were performed before the experimental trials. To control for confounding variables, surface area varied on half of the trials, along with dot size. Attention to numerosity was thus ensured. Panamath generated an estimate of ANS acuity (*w*), based on accuracy at each ratio.

#### Symbolic number comparison

Two digits were simultaneously displayed on the computer screen. The task was to decide, as quickly as possible without making any errors, which of the two digits was the numerically larger one. Prior to each digit pair a fixation cross was displayed in the center of the screen for 1000 ms. After being presented with a pair of digits, the child responded by pressing the key corresponding to the corresponding side of the screen (A-key for the left numeral and the L-key for the right numeral). The digits were visible until the child responded. One-digit numbers and two-digit numbers were presented in two separate blocks, starting with the one-digit block. Two distances between the digits were used: 1 (e.g., 5 vs. 6) and 4–5 (e.g., 1 vs. 6 and 3 vs. 7). Sixteen unique pairs of digits were presented in the one-digit block and each digit pair was presented twice in a reversed position (e.g., 2 vs. 3 and 3 vs. 2) resulting in a total of 32 trials for the one-digit block. The following pairs were used and mirrored in the one-digit block: 1–2, 2–3, 3–4, 4–5, 5–6, 6–7, 7–8, 8–9, 1–6, 2–7, 3–8, 4–9, 1–5, 2–6, 3–7, and 4–8. The following 32 comparison pairs were used in the two-digit block: 21–22, 46–47, 68–69, 86–87, 34–33, 52–51, 74–73, 92–91, 36–37, 58–59, 76–77, 98–99, 29–28, 44–43, 62–61, 84–83, 31–36, 54–59, 71–76, 94–99, 28–23, 47–42, 68–63, 87–82, 21–26, 44–49, 61–66, 84–89, 39–34, 57–52, 79–74, and 97–92. Response times for correct responses and response accuracy were used as dependent measures.

#### Subitizing and enumeration

Arrays of randomly arranged black dots from 1 to 8, with a diameter of 9 mm, were displayed on the computer screen. The child was instructed to state the number of dots displayed on the screen, as quickly as possible without making any errors. The dots were visible until the participant gave a response. A timer controlled by SuperLAB 4.5 software started at the onset of the problem and was stopped when the experimenter pressed the mouse button after the child had given an oral response to the problem. The screen was empty for 1000 ms prior to each problem. A total of 24 problems were presented randomly, that is, each number was presented three times. During the task, the experimenter continuously checked the child's answers, registering each error. Response time was used as the dependent measure. Two measures were created (1–3; 5–8) to determine subitizing and enumeration range. For each child, a mean response time (correct responses only) was calculated for the two measures.

#### Time discrimination

A prospective two-interval time discrimination paradigm was used to measure time perception. The reference stimulus, a red ball, was presented on the center of the computer screen for 3000 ms. After a blank screen was presented for 500 ms, the target stimulus, a blue ball, appeared and remained visible between 1500 and 6000 ms. The task was to determine which of the two stimuli was presented the longest. The child was told to press the corresponding (color-coded) button, the “a”-key was marked with red and “*”-key was marked with blue, after the target stimulus disappeared and was replaced by a response screen. The reference stimulus was fixed at 3000 ms, whereas the target stimulus varied to correspond to four different ratio “bins” (2.0, 1.33, 1.25, 1.20), across 60 trials. Prior to the task, the child was instructed not to use any counting strategies. The number of correct responses was the dependent measure on this task.

#### Mental rotation

The stimuli consisted of alphabetic letters, one letter per item. The test contained 16 items, where the reference was located on the left side accompanied by four comparison stimuli located on the right side adjacent to the target. The comparison stimuli always consisted of two “correct” and two “incorrect” letters. The primary task was to identify the two matching letters, which prompted a mental rotation, and respond by marking them with a pen. Inverted instances of the target (i.e., visually mirrored) were used as incorrect comparison stimuli. All comparison stimuli were rotated only in the picture-plane and in one of six rotation angles (45°; 90°; 135°; 225°; 270°; 315°). The child had to mark both correct comparison stimuli to obtain a point for each item, yielding a maximum score of 16. The child was allowed 2 min to perform the task.

### General procedure

All testing was performed individually over the course of three to four sessions, lasting 30–45 min per session. The total test time for each child was approximately 120–130 min. Instructions were given orally, read aloud from a printed manuscript to ensure that every child was given identical information. At least one practice trial was completed for each task following instructions, in order to eliminate misunderstandings. The computer-administered tasks were run on an Apple Power Mac™ laptop, running SuperLab PRO 4.5 software.

## Results

**Figure 1:**
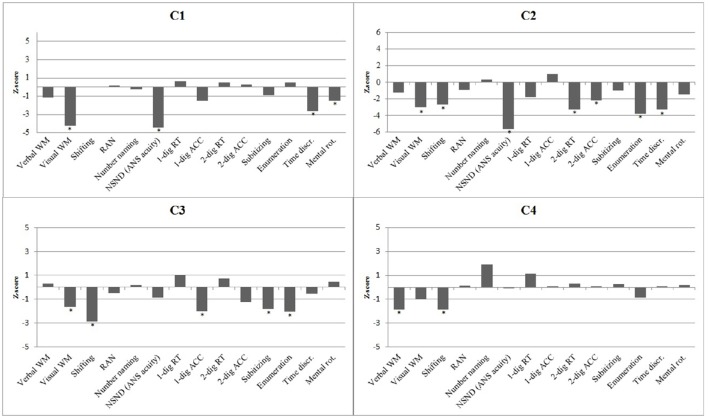
**A histogram of the performances (*z*-scores) of the four cases with DD on domain-general cognitive processing and magnitude processing**. Statistically significant impairments are shown with asterisk.

The performances of the four cases were evaluated in relation to the norm groups by performing a *t*-test for single case methodology (Crawford and Howell, 1998; Crawford and Garthwaite, 2002) for each task. Table 3 shows the raw scores (*t*-score) of the four cases on the domain-general cognitive tasks, the number processing tasks, the time processing task, and the spatial processing task along with descriptive statistics for the norm groups. Provided in Figure 1 is a visual presentation, in the form of a histogram of *z*-scores, of the performances of each case.

**Table 3:** **Performance (*t*-score) of the four cases of DD on domain-general cognitive processing, number processing, time processing, and spatial processing**.

**Task**	**C1 Boy grade 2**	**C2 Girl grade 3**	**C3 Girl grade 2**	**C4 Boy grade 2**	**Norms grade 3**	**Norms grade 2**
Verbal working memory	3 (1.13)	3 (–1.03)	4 (0.32)	2.5 (–1.86)^*^	3.98 (0.76)	3.78 (0.69)
Visual working memory	0 (–4.19)^**^	0 (–3.01)^*^	2 (–1.64)^*^	2.5 (–1.01)	3.36 (1.11)	3.29 (0.78)
Shifting (trail-making)	111 (–0.02)	193 (–2.69)^**^	270 (2.85)^*^	214 (1.84)^*^	71 (45)	112 (55)
Color naming	58 (–0.14)	68 (0.93)	67 (0.49)	58 (–0.14)	55 (13.95)	60 (14.33)
Number naming	194 (0.22)	96 (–0.34)	174 (–0.18)	90 (–1.88)^*^	107 (32)	183 (49)
NSND (Weber fraction) ^†^	0.61	0.72 (5.67)^**^	0.29	0.22	0.21 (0.09)	
1-digit comparison (RT)	1.10 (–0.63)	1.84 (1.76)^*^	0.99 (–0.99)	0.95 (–1.13)	0.96 (0.50)	1.29 (0.30)
1-digit accuracy	27 (–1.46)	32 (0.97)	26 (–1.98)^*^	30 (0.09)	30.24 (1.81)	29.83 (1.92)
2-digit comparison (RT)	1.67 (–0.51)	2.70 (3.29)^**^	1.55 (–0.73)	1.77 (–0.34)	1.35 (0.41)	1.96 (0.56)
2-digit accuracy	28 (–0.26)	26 (–2.18)^*^	25 (–1.23)	30 (0.39)	30.33 (1.98)	28.80 (3.06)
Subitizing 1-3	1.42 (0.87)	1.58 (1.00)	1.65 (1.82)^*^	1.14 (-0.29)	1.26 (0.32)	1.21 (0.24)
Enumeration 5-8	3.40 (–0.37)	6.11 (3.79)^**^	5.03 (2.07)^*^	4.28 (0.86)	3.38 (0.72)	3.74 (0.62)
Time discrimination	25 (–2.62)^*^	21 (–3.30)^**^	38 (–0.57)	42 (0.07)	43.44 (6.78)	41.58 (6.28)
Mental rotation	2 (–1.88)^*^	5 (–1.47)	10 (0.44)	9 (0.32)	10.76 (3.92)	8.16 (4.20)

*NSND, Non-symbolic number discrimination*.

* *p < 0.05*.

** *p < 0.001*.

† *Norms from the Panamath test software for 8 year olds 10th percentile: Weber fraction = 0.57*.

### Assessment of domain-general cognitive abilities

C1 had an impaired visuospatial working memory, *t*_(65)_ = −4.19, *p* < 0.001, whereas his verbal working memory was within the normal range, *t*_(291)_ = −1.13, *p* = 0.153. He also performed within the normal range on the trail-making task, *t*_(65)_ = −0.02, *p* = 0.493, tapping the executive function of shifting ability. He displayed normal processing speed on the color naming and number naming tasks (*p*'s > 0.412).

C2's performances suggest that she had impaired visuospatial working memory, *t*_(114)_ = −3.01, *p* = 0.002, and a defective shifting ability, *t*_(52)_ = 2.69, *p* = 0.005, whereas her general processing speed, tapped by the color naming task and the number naming task was within normal range (*p*'s > 0.177).

C3′s performance pattern indicated deficits in visuospatial working memory, *t*_(65)_ = −1.64, *p* = 0.053, and (executive) shifting ability, *t*_(65)_ = 2.85, *p* = 0.003. On the other hand, her verbal working memory and processing speed of numbers and color names were intact (*p*'s > 0.323).

C4 showed poor verbal working memory ability, *t*_(291)_ = −1.85, *p* = 0.033 and defective shifting ability, *t*_(65)_ = 1.84, *p* = 0.035. In contrast, his visuospatial working memory ability, *t*_(65)_ = −1.01, *p* = 0.159) processing speed of numbers, *t*_(65)_ = −1.88, *p* = 0.032 (indicating superior performance), and color names, *t*_(291)_ = −0.14, *p* = 0.445) showed no signs of impairment.

### Assessment of number, time, and spatial processing

C1's *w*-score (*w* =.61, *z* = −4.44) for the non-symbolic number discrimination task suggest a deficit in the ANS. C1 performed within the normal range on all other number processing tasks (*p*'s > 0.074). C1 also performed poorly on the time discrimination task, *t*_(65)_ = −2.62, *p* = 0.006) and the mental rotation task, *t*_(292)_ = −1.88, *p* = 0.031, indicating impaired temporal and spatial processing.

Similar to C1, C2 displayed severe problems with the non-symbolic number discrimination task, *t*_(273)_ = 5.66, *p* < 0.001, the time discrimination task, *t*_(114)_ = −3.30, *p* < 0.001, and slightly so on the mental rotation task, *t*_(273)_ = −1.47, *p* = 0.072. C2 also performed poorly on the RT measure of the single-digit comparison task, *t*_(273)_ = 1.76, *p* = 0.040, and the two measures of the double-digit comparison task [RT: *t*_(273)_ = 3.29, *p* < 0.001; accuracy: *t*_(273)_ = 2.18, *p* = 0.015]. In addition, C2's ability to enumerate 5–8 dots quickly was impaired, *t*_(273)_ = 3.79, *p* < 0.001, but not the ability to subitize 1–3 dots, *t*_(273)_ = 1.00, *p* = 0.160. C2's performance pattern showed an overall number processing deficit and an impaired ability to process temporal and spatial information.

C3 obtained low accuracy scores on the single-digit comparison task, *t*_(65)_ = −1.98, *p* = 0.026, and displayed slow performance on the subitizing, *t*_(65)_ = 1.82, *p* = 0.034, and enumeration, *t*_(65)_ = 2.07, *p* = 0.022, measures. In contrast, C3's performances on the non-symbolic number discrimination task, the time discrimination task, and the mental rotation task were within the normal range (*p*'s > 0.144). The presentation format of the subitzing task and enumeration task is non-symbolic, but responses prompt a vocal response in the form of number words. Exhibiting impaired performance on these tasks while showing intact performance on number discrimination together indicates that her difficulties were primarily in symbolic processing.

C4 displayed normal performance on all number processing tasks. Furthermore, his performance on the time discrimination task and the mental rotation task were within normal range (*p*'s > 0.376). Thus, C4 did not appear to have any deficits related to the processing of numerical, spatial and temporal information.

## Discussion

The aim of the present study was to further understand the heterogeneity of DD, by testing the main core deficit accounts in relation to four cases of DD. The ATOM model, that a general magnitude-processing deficit underlies DD, was also tested (cf. Skagerlund and Träff, 2014). The question was whether all four cases displayed similar cognitive deficit profiles consistent with the same or different core deficit accounts. The latter outcome would suggest that DD originates from multiple deficits. The four cases will now be discussed in relation to the different accounts.

### C1–magnitude processing subtype

The deficit profile of C1 entails impaired non-symbolic number processing (Price et al., 2007; Landerl et al., 2009; Mussolin et al., 2010; Piazza et al., 2010; Mazzocco et al., 2011a; Mejias et al., 2012), and impaired temporal and spatial processing. These deficits indicate that he has not only a defective ANS, but also a general magnitude processing deficit, as previously reported by Skagerlund and Träff (2014) in a group of fourth graders with DD (see also Ashkenazi and Henik, 2010; Vicario et al., 2012; Moll et al., 2014). C1's profile also encompasses a defective visuospatial working memory capacity (cf. Andersson, 2010; Raghubar et al., 2010). Given that C1 showed normal shifting ability and verbal working-memory, the apparent deficit in visuospatial working-memory is likely caused by a general magnitude processing deficit, consistent with the ATOM model. Although working-memory functionality in general may be intact in C1, he may have problems in encoding and retaining spatial information in working-memory. Visuospatial working memory processing involves the right fronto-parietal network, comprising of the right IPS and the right inferior frontal gyrus (Rotzer et al., 2009). The inferior frontal gyrus may in this case be intact, but receives impoverished spatial information from the IPS because of a magnitude processing deficit.

Previous hypotheses have stated that due to shared neural resources between quantity representations and other continuous magnitude processes, a deficit in quantity processing should affect other dimensions as well, such as time and space (Feigenson, 2007). Time perception has been attributed to neural processing in the parietal cortex and insula (Lewis and Miall, 2003; Wiener et al., 2010), whereas Kucian et al. (2007) found that mental rotation tasks are subserved by cortical substrates in the IPS. The intraparietal cortex is also known to be involved in visual attention (Klingberg et al., 2002), which could explain the concomitant and severe visuospatial working-memory deficit shown by C1. Visual attention may project information via dorsal visual stream to frontal areas to be encoded in working-memory, in this case a set of dots. As C1 did not show impairments in verbal working memory, one tentative interpretation is that the deficit is limited to visuospatial information sub-served by occipitoparietal visual processing. The posterior IPS has structural connections with extrastriate visual areas (Uddin et al., 2010), indicating that this cortical circuitry may be susceptible to abnormalities. They could subsequently hamper visuospatial processing. Uddin et al. (2010) suggested that the posterior IPS may play a role in transforming symbolic and non-symbolic numerical information to spatial and semantic representations of quantity. Additionally, the anterior IPS has structural connections with insula (Uddin et al., 2010), which is involved in time perception (Lewis and Miall, 2003) and might be part of a structural network for magnitude processing in general.

In sum, the cognitive profile of C1 suggests primary DD with a magnitude processing deficit that impedes mathematical reasoning. Neurocognitive correlates would likely be traced to the IPS and the dorsal visual stream.

### C2–magnitude processing and domain-general subtype

C2 has a complex deficit profile; a general number processing deficit that involved non-symbolic and symbolic number processing and enumeration ability, but intact subitizing ability. However, it should be noted that the dots in the subitizing task were visible until the participants gave their response, limiting the inferential power from this task. However, poor subitizing ability should shine through in terms of longer RT's even if the child was counting the dots instead of subitizing them.

Nevertheless, this pattern is consistent with the defective ANS account (e.g., Price et al., 2007; Piazza et al., 2010; Mejias et al., 2012). Another interesting aspect of C2's profile is the impaired ability to process temporal and spatial information, like C1, which in combination with her number processing deficits suggest a general magnitude processing deficit. C2's deficit profile also points to domain-general cognitive deficits, such as impaired visuospatial working memory (Raghubar et al., 2010) and a defective executive function of shifting (McLean and Hitch, 1999; van der Sluis et al., 2004).

Given C2's widespread cognitive impairments, it is hard to point to any singular cortical area that might be dysfunctional. It is likely that a few key structural connections or cortical loci are dysfunctional, which in turn cascade into large-scale cognitive deficits of number processing as well as domain-general processing. Also, it is likely that C2 has the same cortical deficits as C1, with additional deficits pertaining to frontal areas of the brain.

In sum, C2 likely suffers from primary DD characterized by a combined magnitude processing deficit and domain-general cognitive impairment.

### C3–access deficit and domain-general subtype

C3′s deficit profile is characterized by defective symbolic number processing, but intact non-symbolic number processing (cf. Rousselle and Noël, 2007; De Smedt and Gilmore, 2011). This is consistent with the access deficit hypothesis and possibly the defective OTS hypothesis, as C3's subitizing ability was impaired (Desoete and Grégoire, 2006; Moeller et al., 2009; Andersson and Östergren, 2012). However, it should be noted that, as in the case of C2, the interpretations of the performance on the subitizing task are limited given that the presentation time of the stimuli is tied to the responses. Although we encouraged the children to respond as fast as possible, we cannot exclude the possibility that C2 and C3 used a counting process instead of a subitzing process.

Her deficit profile also involves deficits in visuospatial working memory and (executive) shifting ability. C3 highlights the likely dissociation between non-symbolic approximate number processing (taxing the ANS), exact enumeration and subitizing. The results indicate that the magnitude processing system is intact overall, given that C3 had an unimpaired ANS and normal processing of time and space.

In contrast, C3 displayed problems with several tasks relying heavily on neurocognitive processing and neural loci in the frontal areas of the brain. Visuospatial working memory processing involves a right fronto-parietal network, comprising of the right IPS and the right inferior frontal gyrus. As C3 showed an unimpaired ANS, which relies on IPS bilaterally, one interpretation is that either (a) the cortical dysfunction is circumscribed to inferior frontal gyrus or (b) a white matter connectivity issue impedes information processing in the fronto-parietal network. Support for the latter comes from the impaired performance on the symbolic number processing task. Although C3 had no problems with rapidly naming digits, she did struggle with selecting which number is the highest in the symbolic number comparison task. This suggests that C3 suffers from an access deficit that impedes the accurate mapping of symbols to their underlying quantity representation. The underlying neural dysfunction responsible for the access deficit is unclear. C3 could rapidly decode digits and express them verbally, which may indicate that the ability to process complex visual stimuli subserved by the fusiform gyrus, lingual gyrus, and hippocampal regions is intact. One tentative interpretation is that frontal regions, such as dorsolateral prefrontal cortex and inferior frontal gyrus, is implicated during access to the semantic referents (Nieder, 2009), and may be involved during active counting due to effortful working memory demands.

Throughout ontogenetic development, typically developing children show increased white matter density in the frontal lobes, suggesting a maturation of the fronto-parietal network (Ranpura et al., 2013). Children with DD, however, do not show the same significant increase in white matter volume, which may indicate that DD may be partly due to rudimentary associations between symbols and their underlying magnitude representations (Rykhlevskaia et al., 2009). Given that C3 also show extensive deficits in working-memory capacity and shifting ability, partly relying on the prefrontal cortex, it is likely that frontal dysfunctions inhibit active and explicit processing of numerical content. This would also explain C3's apparent difficulty with subitizing and enumeration, since the task requires verbal answers.

Taken together, C3 may have primary DD with an access deficit subtype conjointly with a domain-general cognitive impairment.

### C4–domain-general subtype

The deficit profile of C4 is restricted to poor verbal working memory (cf. Raghubar et al., 2010), and defective shifting ability (cf. McLean and Hitch, 1999; van der Sluis et al., 2004). Thus, corresponding with the domain-general cognitive deficit account of DD. Shifting ability and verbal working-memory rely heavily on the cognitive control network or *salience network* (Menon and Uddin, 2010; Engström et al., 2013), comprising of anterior cingulate cortex (ACC), insula, and dorsolateral prefrontal cortex. Given that insula and ACC are important in magnitude processing as well, especially during time processing, these areas are likely intact in C4 due to the normal magnitude processing capabilities. Thus, C4 may have problems in encoding and retaining verbal information in working-memory during mathematical reasoning, which may hamper the ability to maintain control of intermediate steps during calculation and problem-solving.

**Figure 2:**
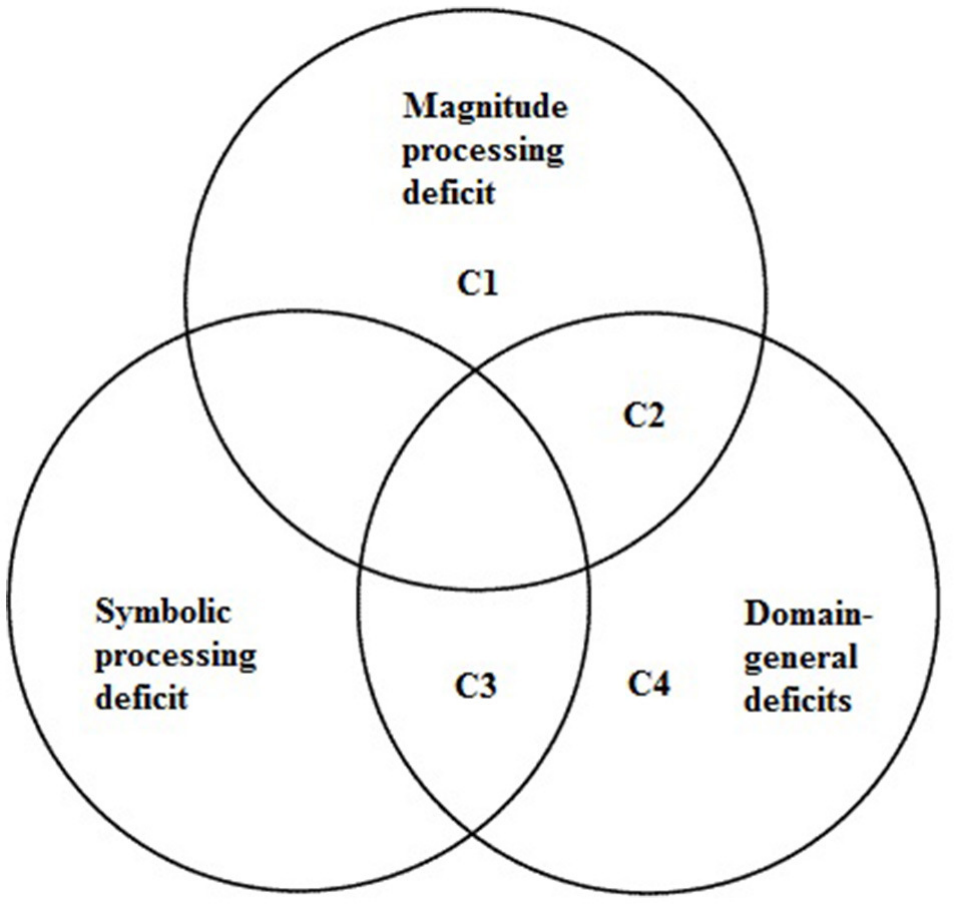
**A diagram of the different deficits and the resulting profile of the children**.

In contrast to the previous cases, C4 cannot be characterized as having primary DD, as the mathematical difficulties are likely caused by a domain-general cognitive impairment; hence, C4 likely has secondary DD (Kaufmann et al., 2013). See Figure 2 for a diagram of the different deficit profiles.

## Concluding discussion

The present study clearly demonstrates that only relying on traditional studies utilizing group comparisons is not sufficient for exploring the heterogeneity of DD. Collapsing all individuals into one, supposedly, homogeneous group may obfuscate important interindividual variability. The different cognitive deficit profiles of the present cases provide strong support for a multideficits account of DD, further corroborating findings reported by Bartelet et al. (2014), Iuculano et al. (2008) and Skagerlund and Träff (2016). Furthermore, not only did the cases display profiles consistent with different accounts, three of the cases displayed profiles consistent with more than one account (cf. Andersson and Östergren, 2012). The profiles of cases C1 and C2 are consistent with the ANS deficit hypothesis, the general deficit account and, interestingly, the general magnitude-processing deficit account.

C3 had an access deficit in combination with a general cognitive deficit. C4, however, only suffered from domain-general cognitive deficits. These patterns of deficits are important as they indicate that DD can originate from different constellations of deficits at an individual level. This is important to consider when planning and executing interventions, because it might be necessary to target several different cognitive skills.

It was somewhat unexpected to find that all four cases displayed concomitant deficits in domain-general abilities in tandem with any deficit in core number ability. More specifically, all cases showed either verbal and/or visuospatial working memory deficits (Raghubar et al., 2010). In addition, all cases except C1, display executive function impairment in shifting ability (McLean and Hitch, 1999; van der Sluis et al., 2004). Thus, none of the cases showed a deficit circumscribed to number processing alone, which is the defining feature of DD according to some researchers (Rubinsten and Henik, 2009). In contrast, isolated domain-general cognitive deficits are identified in one case (C4) in which no number processing deficits are manifest.

Although promising, the distinction between primary and secondary DD proved to be not entirely unequivocal. Kaufmann et al. (2013) proposed that secondary DD should be used if “*numerical/arithmetic dysfunctions are entirely caused by non-numerical impairments (e.g., attention disorders)*” (p. 4). We agree with the authors and believe that the definition is sound, but it proved hard to disentangle the domain-general processes from numerical processes even at an individual level. For example, C3 displayed a profile suggestive of an access deficit. However, given the coexisting deficits in executive functions and visuospatial working-memory, it cannot be ruled out that these deficits play a causative role in the apparent number processing deficit. Executive functions may have affected performance on the symbolic number comparison task during the task situation itself, or executive functions may have hampered the mapping between symbols over the long-term throughout development. By factoring in that C3 has normal reading skills, involving symbols and their semantic referents, and performed normally on the non-symbolic ANS task involving selection of either of two alternatives, it is plausible that C3 has a “genuine” deficit in number processing involving symbols. Nevertheless, it is unclear whether C3 can be said to suffer from an access deficit and fit the profile of Rousselle and Noël (2007) given the concomitant domain-general deficits. However, it is likely that the mathematical difficulties of C3 cannot be attributed *entirely* to non-numerical factors, which is the defining feature of secondary DD. That brings us to whether C3 suffers from primary DD, which is defined as a “*heterogeneous disorder resulting from individual deficits in numerical or arithmetic functioning at behavioral, cognitive/neuropsychological and neuronal levels”* (Kaufmann et al., 2013, p. 4). The access deficit shown by C3 fits this definition nicely, but it is unclear to what extent domain-general abilities can contribute to these defining characteristics. One strict interpretation of primary DD would be that one has to show *only* number processing deficits while displaying no domain-general deficits whatsoever. A more lenient interpretation would be that a number processing deficit is both a necessary and sufficient criterion of primary DD, in which case number processing deficits and domain-general deficits can coexist. This ambiguity led us to interpret C3 as having primary DD with an access deficit subtype with concurrent domain-general subtype. We welcome the distinction between primary DD and secondary DD, but we urge for further discussion about the heterogeneity and the defining features of each of these. Further work should also address the challenges in disentangling different cognitive processes and deficits when assessing children at risk of developing DD so that appropriate interventions can be implemented.

A key finding is that two of the cases (C1, C2) displayed a general magnitude-processing deficit. In view of the ATOM model (Walsh, 2003; Bueti and Walsh, 2009), the deficit profiles of C1, C2, and C3 suggest that children with DD suffering from a deficit in the innate ANS (C1; C2) might also have impaired spatial and temporal processing skills. They should therefore be regarded as having a general magnitude processing deficit. The child that showed an access deficit, C3, however showed impaired symbolic number processing but intact magnitude processing skills in all dimensions. This dissociation suggests that symbolic number processing is connected to dimension-specific number processes and not to the partly shared general magnitude system.

Using a comprehensive test battery to investigate the cognitive profiles in depth, we can make nuanced interpretations about specific weaknesses. For example, C1 showed weakness of the ANS, magnitude processing and visuospatial-working memory capacity, consistent with two prominent hypotheses about DD. One advantage of our methodological approach is that we can triangulate cognitive weaknesses. C1 showed impaired visuospatial working memory capacity, and if a traditional group analysis with a single specific hypothesis about the importance of visuospatial working memory ability had been used, a quite heterogeneous group showing impaired visuospatial working-memory processing might have been found. However, the individuals in this group might have displayed this impairment for different underlying reasons. Some individuals might have genuine neurocognitive issues pertaining to deficient activity in frontal areas of the brain, whereas others may have a dysfunction in a precursor process in IPS involving magnitude information. The dysfunctional magnitude process may then propagate impoverished information via dorsal stream to frontal areas of the brain responsible for subsequent working-memory processes. By administering a comprehensive set of tasks, we tentatively suggest that the apparent difficulties in visuospatial working memory displayed by C1 is a by-product of another process, hence getting a more nuanced and elaborate understanding of the entire cognitive profile of an individual with DD. Although making matters more complex, and naturally subject to both replication and verification using imaging data such as fMRI and DTI, we want to show that DD is a complex condition and we should treat it as such. This requires a multilevel approach, and we cannot rely solely on group analyses that measure single constructs collapsed across numerous individuals at a single point in time. We acknowledge the tentative nature of our neurocognitive hypotheses, but we hope that these can inform and guide future neuroimaging studies that broaden our understanding of this very complex learning disorder.

## Ethics statement

Regional Ethics Committee in Linköping, Sweden. Reference number: Dnr 2011/58-31. After schools had agreed to participate, the parents filled out a consent form that was sent home. This study and procedure has been approved by the Regional Ethics Committee in Linköping, Sweden, (reference number: Dnr 2011/58-31). Informed consent was given by parent or other legal guardian.

## Author contributions

UT conceived of the study and contributed to the overall study design and statistical analyses. LO contributed to manuscript preparation and analysis. RÖ participated in the design phase and planning of the tasks used. KS contributed to the design, making analyses, and with manuscript preparation.

## Funding

This research was supported by grants from the Swedish Research Council for Health, Working Life and Welfare (2008-0238 and 2010-0078) awarded to UT.

### Conflict of interest statement

The authors declare that the research was conducted in the absence of any commercial or financial relationships that could be construed as a potential conflict of interest.
